# AMPD1 regulates mTORC1-p70 S6 kinase axis in the control of insulin sensitivity in skeletal muscle

**DOI:** 10.1186/s12902-015-0010-9

**Published:** 2015-03-27

**Authors:** Andreas AK Tandelilin, Tetsuaki Hirase, Athanasius W Hudoyo, Jidong Cheng, Keiko Toyama, Hiroko Morisaki, Takayuki Morisaki

**Affiliations:** Department of Bioscience and Genetics, National Cerebral and Cardiovascular Center Research Institute, 5-7-1 Fujishirodai, Suita, Osaka 565-8565 Japan; Department of Molecular Imaging in Cardiovascular Medicine, Osaka University Graduate School of Medicine, Suita, Osaka Japan; Department of Molecular Pathophysiology, Osaka University Graduate School of Pharmaceutical Sciences, Suita, Osaka Japan; Present address: Department of Internal Medicine, The First Affiliated Hospital of Shantou University Medical College, Shantou, Guangdong 515031 P R China

**Keywords:** AMPD1, AMPK, Akt, p70 S6 kinase, mTORC1, PGC1

## Abstract

**Background:**

Insulin resistance triggered by excess fat is a key pathogenic factor that promotes type 2 diabetes. Understanding molecular mechanisms of insulin resistance may lead to the identification of a novel therapeutic target for type 2 diabetes. AMPD1, an isoform of AMP deaminase (AMPD), is suggested to play roles in the regulation of glucose metabolism through controlling AMP-activated protein kinase (AMPK) activation. We reported that the diet-induced insulin resistance was improved in AMPD1-deficient mice compared to wild type mice. To further delineate this observation, we studied changes of insulin signaling in skeletal muscle of wild type (WT) and AMPD1-deficient mice.

**Methods:**

Phosphorylation levels of kinases and expression levels of mTOR components were quantified by immunoblotting using protein extracts from tissues. The interaction between mTOR and Raptor was determined by immunoblotting of mTOR immunoprecipitates with anti-Raptor antibody. Gene expression was studied by quantitative PCR using RNA extracted from tissues.

**Results:**

Phosphorylation levels of AMPK, Akt and p70 S6 kinase in skeletal muscle were higher in AMPD1-deficient mice compared to WT mice after high fat diet challenge, while they did not show such difference in normal chow diet. Also, no significant changes in phosphorylation levels of AMPK, Akt or p70 S6 kinase were observed in liver and white adipose tissue between WT and AMPD1-deficient mice. The expression levels of mTOR, Raptor and Rictor tended to be increased by AMPD1 deficiency compared to WT after high fat diet challenge. AMPD1 deficiency increased Raptor-bound mTOR in skeletal muscle compared to WT after high fat diet challenge. Gene expression of peroxisome proliferator-activated receptor-γ coactivator 1α and β, downstream targets of p70 S6 kinase, in skeletal muscles was not changed significantly by AMPD1 deficiency compared to the wild type after high fat diet challenge.

**Conclusion:**

These data suggest that AMPD1 deficiency activates AMPK/Akt/mTORC1/p70 S6 kinase axis in skeletal muscle after high fat diet challenge, but not in normal chow diet. These changes may contribute to improve insulin resistance.

## Background

Insulin resistance that is characterized by reduced sensitivity and response to insulin action of insulin-sensitive organs is an important pathogenic factor for type 2 diabetes triggered by excess nutrient intake, in addition to impaired insulin secretion caused by pancreatic β cell dysfunction [1]. Among insulin-sensitive organs, skeletal muscle is a major contributor to glucose tolerance through regulating glucose uptake by glucose transporter [2]. Therefore, understanding the molecular mechanism that regulates insulin sensitivity in skeletal muscle would lead to the identification of novel therapeutic target for type 2 diabetes.

Metabolic stress changes cellular adenine nucleotide levels and resultantly modifies cellular response. AMP deaminase (AMPD) catalyses the deamination of AMP into inosine monophosphate (IMP) and determines cellular AMP levels [3]. Cellular AMP level is a crucial determinant for the activation of AMP-activated protein kinase (AMPK) that is a key regulator of energy, glucose and lipid metabolism and possesses anti-diabetic property [4]. Accordingly, AMPD is considered to participate in the regulation of AMPK. AMPD has three isoforms designated as AMPD1, 2 and 3. AMPD1 is highly selectively expressed in skeletal muscle, of which mutations may be involved in human metabolic myopathy [3]. Variation of *AMPD1* gene is reportedly associated with higher insulin clearance in human [5]. A lower incidence of type 2 diabetes in AMPD1 deficient human subjects is also demonstrated [6]. Therefore, we have studied roles of AMPD1 in the regulation of glucose metabolism using AMPD1 deficient mice made by the gene targeting [7,8]. We demonstrated that AMPD1 deficient mice show augmented glucose tolerance and attenuated insulin resistance under high fat diet feeding triggering insulin resistance [8].

In this study, we investigated insulin signaling in insulin-sensitive organs from control wild type C57BL6 mice and AMPD1-deficient mice fed with normal chow and high fat diet in order to further analyze the underlying mechanism for augmented glucose tolerance and attenuated insulin resistance induced by AMPD1 deficiency under high fat diet feeding. We studied the activation of kinases that participate in insulin signaling by assessing their auto-phosphorylation levels. Since we demonstrated enhanced AMPK phosphorylation in skeletal muscle by AMPD1 deficiency, we tried to analyze Akt phosphorylation and the activation of mammalian mTOR complex1 (mTORC1), which is a crucial link of AMPK and Akt in regards to insulin signaling. Also, we investigated p70 S6 kinase [9] as the downstream target of these molecules. Therefore, we studied AMPK/Akt/mTORC1/p70 S6 kinase axis in skeletal muscle after high fat diet challenge in AMPD1-deficient mice to show the impact of AMPD1 on insulin resistance.

## Methods

### Animals

C57BL/6 control mice and AMPD1 deficient (*Ampd1*−/−) mice generated as described previously [7,8] housed in a controlled SPF environment with a 12-hour light–dark cycle and constant temperature (25°C) at 5 weeks old were fed with normal chow diet (CE-2; CLEA Japan, Inc., Tokyo, Japan) or a high-fat diet (HFD32; CLEA Japan, Inc.) with 60% fat calories for 12 weeks. Then, mice were fasted for 17 hours and sacrificed under anesthesia with isoflurane.

All animal experiments approved by the Committee on Animal Research of National Cerebral and Cardiovascular Center were performed according to the guidelines for the protection of experimental animals of National Cerebral and Cardiovascular Center.

### Immunoblotting

Tissues were homogenized into RIPA buffer containing 10 mM Tris pH7.4, 1% NP40, 0.1% sodium deoxycholate, 0.1% SDS, 0.15 M NaCl, 1 mM EDTA, cOmplete ULTRA protease inhibitor cocktail (Roche Diagnostics, Mannheim, Germany) and PhosSTOP phosphatase inhibitor cocktail (Roche Diagnostics). After centrifuge, the supernatant of the homogenate was collected and used for protein determination with a BCA Protein Assay Kit (Pierce, Rockford, IL, USA). The supernatant was boiled with Laemmli sample buffer, resolved by one dimensional SDS-PAGE and then electrophoretically transferred to polyvinylidene difluoride membranes using Trans-Blot Turbo System (Bio-Rad Laboratories Inc., Hercules, CA, USA). The membranes were subjected to immunoblotting as described previously with different primary antibodies (anti-AMPKα antibody, #2532; anti-phosphoAMPKα (Thr172) antibody, #2535; anti-Akt antibody, #4691; anti-phospho Akt (Ser473) antibody, #4060; anti-p70 S6 kinase antibody, #2708; anti-phospho p70 S6 kinase (Thr389) antibody, #9234: anti-mTOR antibody, #2983; anti-Raptor antibody, #2280; anti-Rictor antibody, #2114; anti-α − tubulin antibody, #2125; Cell Signaling Technology, Danvers, MA, USA) (8). Signals were developed with an ECL Prime kit (GE Healthcare, Piscataway, NJ, USA) and detected using LAS-1000 Luminescent Image Analyzer (FUJIFILM, Tokyo, Japan). Band intensity was quantified using Image Reader LAS-1000 software (FUJIFILM). Samples from five mice of each group were analyzed.

### Immunoprecipitation

We immunoprecipitated mTOR following a previous report [10]. Tissue was homogenized with a lysis buffer containing 0.3% CHAPS. After centrifuge, the supernatant was collected and used for protein determination with a BCA Protein Assay Kit (Pierce). The input represented 5% of the lysate used for each immunoprecipitation for the normalization with the levels of a housekeeping gene product α-tubulin. Lysates were incubated with anti-mTOR antibody (#2972, Cell Signaling Technology) at 4°C for 1 hour followed by the incubation with Dynabeads Magnetic Beads (Life Technologies, Carlsbad, CA, USA) at 4°C for another 1 hour. Beads were washed with lysis buffer and immunoprecipitates were eluted by boiling with Laemmli sample buffer.

### RNA extraction and quantitative PCR

RNA was isolated from tissues with Isogen II following the manufacturer’s protocol (Nippon gene, Tokyo, Japan). cDNA synthesis was performed using 2 μg total RNA and SuperScript III following the manufacturer’s protocol (Invitrogen, Carlsbad, CA, USA). Real-time PCR products performed with Power SYBR Green Master Mix (Life Technologies) were analyzed using an ABI PRISM 7900 sequence detection system (Applied Biosystems) and normalized with control β-actin mRNA levels. The specific primer sets for the target genes were as follows: PGC-1α, 5′-CAGCCTCTTTGCCCAGATCT-3′ and 5′-CCGCTAGCAAGTTTGCCTCA-3′; PGC-1β, 5′-CTTTGACTCAGCCACGTGCT-3′ and 5′-CGCAGCCAAGAGAGCTTCAT-3′.

### Statistical analysis

Data are expressed as means ± SD. Data were analyzed by parametric ANOVA followed by Tukey’s multiple comparison test. We considered that *P* values smaller than 0.05 were statistically significant.

## Results

We investigated the activation of insulin signaling pathways in which AMPK, Akt and p70 S6 kinase participate in insulin-sensitive tissues such as skeletal muscle, liver and white adipose tissue. Since AMPK, Akt and p70 S6 kinase are activated by upstream kinases in response to stimuli through auto-phosphorylation, we evaluated their phosphorylation levels by immunoblotting.

**Figure 1:**
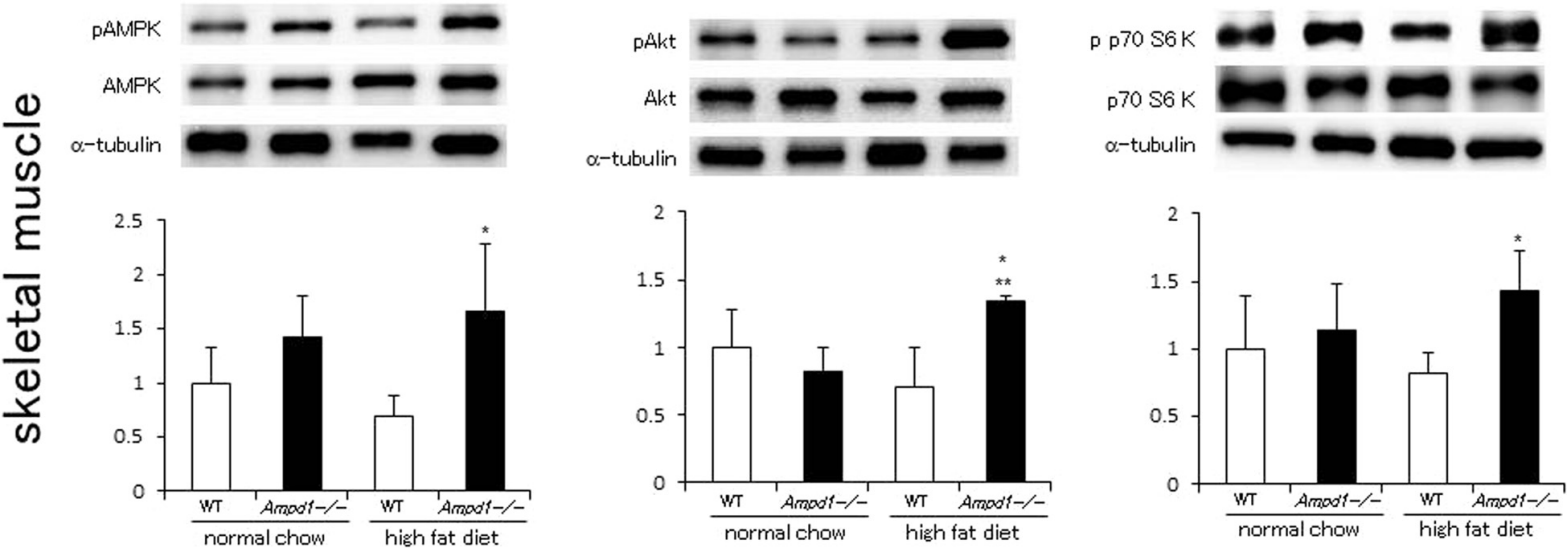
**AMPD1 deficiency significantly augmented phosphorylation of AMPK, Akt and p70 S6 kinase after high fat diet challenge in skeletal muscles.** Protein extracts from gastrocnemius muscle of wild type and AMPD1 deficient mice fed with normal chow and high fat diet were studied by immunoblotting for AMPK/phosphorylated AMPK (pAMPK), Akt/phosphorylated Akt (pAkt) and p70 S6 kinase/phosphorylated p70 S6 kinase (pp70 S6kinase) (*n* = 5 for each group). The ratio of the band intensity for phosphorylated form to that of total form measured as described in Methods was quantified and adjusted with that of α-tubulin. Representative immunoblot images are shown in upper panels. Data shown in lower panels are mean ± SD expressed relative to that of WT mice fed with normal chow. *and **indicate *p* < 0.05 vs. WT mice fed with high fat diet and AMPD1-deficient mice fed with normal chow, respectively.

Phosphorylation level of AMPK tended to elevate by AMPD1 deficiency in mice fed with normal chow diet (left panel, Figure 1). AMPK phosphorylation was significantly enhanced by AMPD1 deficiency in mice fed with high fat diet. Akt phosphorylation was also significantly augmented by AMPD1 deficiency in mice fed with high fat diet, but not with normal chow diet (middle panel, Figure 1). As a downstream of insulin signaling, phosphorylation of p70 S6 kinase was significantly augmented by AMPD1 deficiency in mice fed with high fat diet, but not with normal chow diet (right panel, Figure 1).

**Figure 2:**
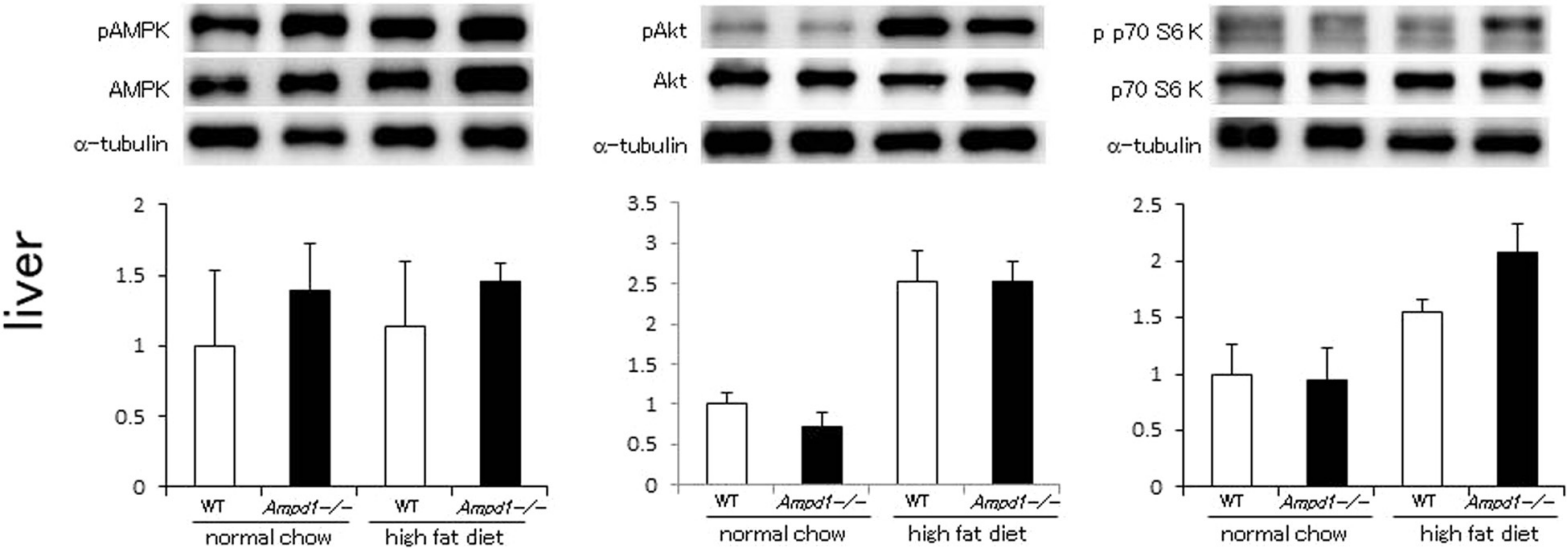
**AMPD1 deficiency did not change phosphorylation levels of AMPK, Akt and p70 S6 kinase after high fat diet challenge in liver.** Protein extracts from liver of wild type and AMPD1 deficient mice fed with normal chow and high fat diet were studied by immunoblotting for AMPK/phosphorylated AMPK (pAMPK), Akt/phosphorylated Akt (pAkt) and p70 S6 kinase/phosphorylated p70 S6 kinase (pp70 S6kinase) (*n* = 5 for each group). The ratio of the band intensity for phosphorylated form to that of total form was quantified and adjusted with that of α-tubulin. Representative immunoblot images are shown in upper panels. Data shown in lower panels are mean ± SD expressed relative to that of WT mice fed with normal chow.

In liver, AMPK phosphorylation was not significantly changed by AMPD1 deficiency either in normal chow diet or under high fat diet challenge (left panel, Figure 2). Akt phosphorylation showed no significant difference between wild type and AMPD1-deficient mice both in normal chow diet and under high fat diet challenge (middle panel, Figure 2). In good accordance with changes in phosphorylation levels of AMPK and Akt, p70 S6 kinase phosphorylation was not significantly changed by AMPD1 deficiency either in normal chow diet or under high fat diet challenge (right panel, Figure 2).

**Figure 3:**
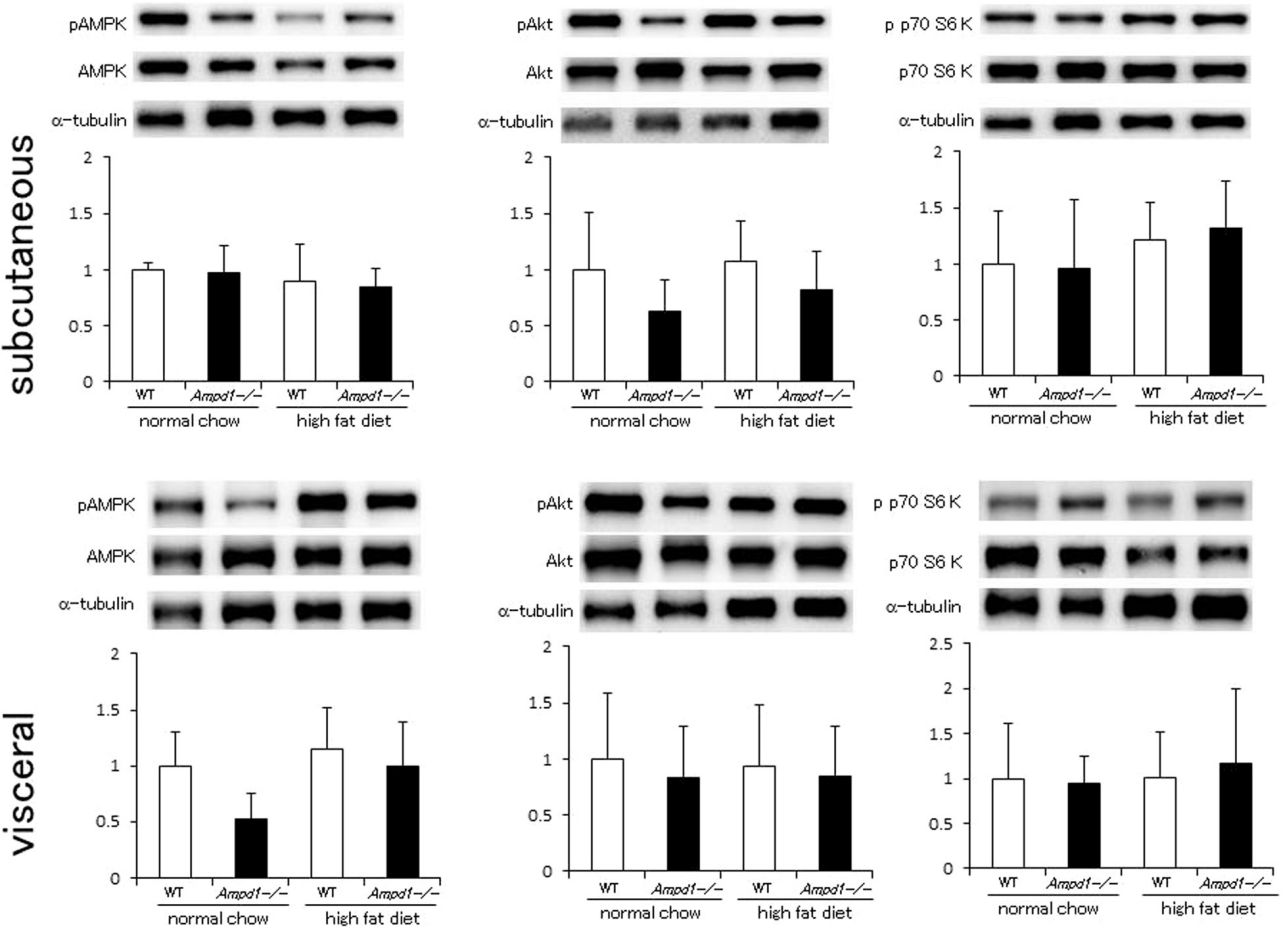
**AMPD1 deficiency did not change phosphorylation levels of AMPK, Akt and p70 S6 kinase after high fat diet challenge in white adipose tissue.** Protein extracts from subcutaneous and visceral adipose tissue of wild type and AMPD1 deficient mice fed with normal chow and high fat diet were studied by immunoblotting for AMPK/phosphorylated AMPK (pAMPK), Akt/phosphorylated Akt (pAkt) and p70 S6 kinase/phosphorylated p70 S6 kinase (pp70 S6kinase) (*n* = 5 for each group). The ratio of the band intensity for phosphorylated form to that of total form was quantified and adjusted with that of α-tubulin. Representative immunoblot images are shown in upper panels. Data shown in lower panels are mean ± SD expressed relative to that of WT mice fed with normal chow.

AMPK phosphorylation demonstrated no significant difference between WT and AMPD1-deficient mice fed with normal chow and high fat diet in subcutaneous adipose tissue (upper left panel, Figure 3). Akt phosphorylation tended to be lower, but not significant in AMPD1-deficient subcutaneous adipose tissue compared to control under normal chow condition or high fat diet feeding (upper middle panel, Figure 3). Phosphorylated p70 S6 kinase levels also showed no significant difference between WT and AMPD1-deficient mice (upper right panel, Figure 3).

Phosphorylation levels of AMPK (lower left panel, Figure 3), Akt (lower middle panel, Figure 3) and p70 S6 kinase (lower right panel, Figure 3) showed no significant difference between WT and AMPD1-deficient mice under normal chow condition and high fat diet feeding in epididymal white adipose tissue that is included in visceral adipose tissue.

These data suggest that AMPD1 deficiency significantly enhances activation of AMPK, Akt and p70 S6 kinase in skeletal muscle under high fat diet, in contrast to normal chow diet condition. AMPD1 deficiency seems not to change activation of AMPK, Akt and p70 S6 kinase in liver or white adipose tissue including subcutaneous and visceral adipose tissue under normal chow and high fat diet. Differential regulation of AMPK/Akt/p70 S6 kinase indicates that skeletal muscle is primary target of the deficiency of AMPD1 which exhibits a highly selective expression in skeletal muscle.

**Figure 4:**
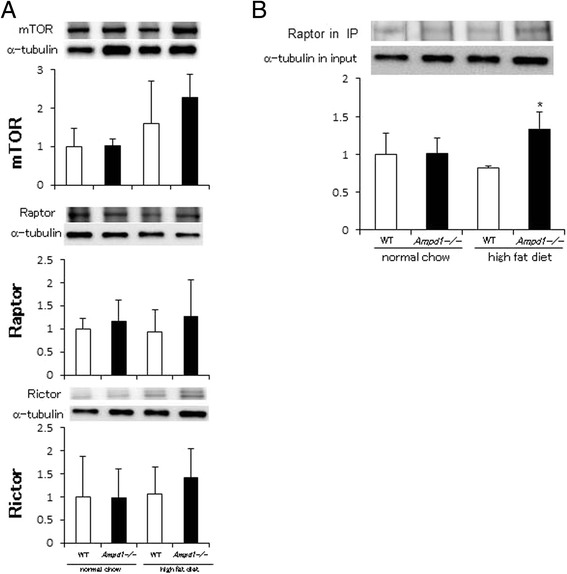
**Promoted mTORC1 formation in skeletal muscle after high fat diet feeding by AMPD1 deficiency. (A)** Protein extracts from gastrocnemius muscle of wild type and AMPD1 deficient mice fed with normal chow and high fat diet were subjected to immunoblotting for mTOR, Raptor and Rictor (*n* = 5 for each group). The band intensity was quantified and adjusted with that of α-tubulin. Representative immunoblot images are shown in upper panels. Data shown in lower panels are mean ± SD expressed relative to that of WT mice fed with normal chow. **(B)** mTOR immunoprecipitates from gastrocnemius muscle were analyzed by immunoblotting with anti-Raptor antibody. The band intensity of mTOR-bound Raptor in immunoprecipitates was adjusted with that of α-tubulin in the input lysates used for immunoprecipitation. Representative immunoblot images of Raptor in mTOR immunoporecipitates and α-tubulin in the input lysates are shown in upper panels. Data shown in lower panel are mean ± SD expressed relative to that of WT mice fed with normal chow (*n* = 5 for each group). *indicates *p* < 0.05 vs. WT mice fed with high fat diet.

mTOR integrates insulin signal mediated by AMPK and Akt and transduces signal to downstream targets including p70 S6 kinase through formation of mTORC1 with Raptor as a positive regulator and mTORC2 with Rictor as a negative regulator [9,10]. Therefore, we compared the expression of mTOR, Raptor and Rictor and the formation of mTORC in skeletal muscle from WT and AMPD1-deficient mice (Figure 4). Protein expression of mTOR, Raptor and Rictor showed no significant difference between WT and AMPD1-deficient mice fed with normal chow, while AMPD1 deficiency tended to increase protein expression of mTOR, Raptor and Rictor in skeletal muscle after high fat diet challenge compared to control (Figure 4A). We quantified the amount of mTORC1 as a positive regulator of mTOR signaling by detecting mTOR-bound Raptor in mTOR immunoprecipitates from lysates of skeletal muscle (Figure 4B). Since mTOR expression levels were changed by AMPD1 deficiency as shown in Figure 4A, we adjusted the band intensity of mTOR-bound Raptor with that of a housekeeping gene product α-tubulin detected in 5% protein amount of the input lysates which were used for immunoprecipitation. mTOR-bound Raptor in skeletal muscle showed no obvious difference between WT and AMPD1-deficient mice fed with normal chow, while AMPD1 deficiency significantly increased mTOR-bound Raptor in skeletal muscle after high fat diet challenge compared to control. These data suggest that AMPD1 deficiency induces the expression of mTORC components and upregulates mTOR signaling by promoting mTORC1 formation in skeletal muscle after high fat diet feeding.

**Figure 5:**
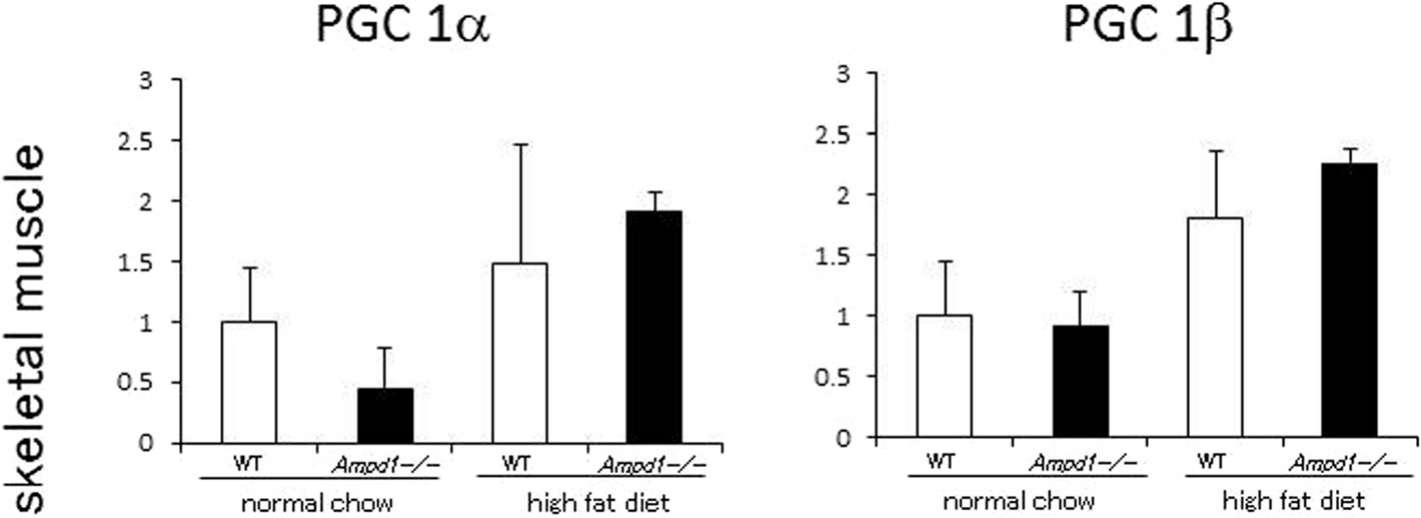
**Effects of AMPD1 deficiency on gene expression of downstream targets of p70 S6 kinase in skeletal muscle.** mRNA expression of PGC1α and β was quantified by real-time PCR using RNA extracted from gastrocnemius muscle of WT and AMPD1 deficient mice fed with normal chow and high fat diet (*n* = 5 for each group). The mRNA expression levels were normalized with respect to β-actin expression. Data shown are mean ± SD expressed relative to that of WT mice fed with normal chow.

As the current study indicates that AMPK/Akt/mTOR/p70 S6 kinase pathway is upregulated in skeletal muscle by AMPD1 deficiency under high fat diet feeding, we studied gene expression of peroxisome proliferator-activated receptor-γ coactivator-1 (PGC1) α and β in skeletal muscle that are downstream targets of mTOR/p70 S6 kinase pathway [11]. PGC1 comprises a family of transcription factor that regulates cellular energy metabolism through mitochondria biogenesis and participates in the control of glucose and lipid metabolism. mRNA expression of PGC1α and β in skeletal muscle showed no significant difference between WT and AMPD1-deficient mice under normal chow diet as well as high fat diet challenge (Figure 5). These data suggest that AMPD1 deficiency induces upregulated gene expression of downstream targets of p70 S6 kinase in skeletal muscle after high fat diet challenge which could contribute to attenuated insulin resistance.

## Discussion

Insulin resistance defines a condition in which tissue sensitivity to insulin action is decreased due to inhibited intracellular insulin signaling [1,2]. Obesity induced by excess nutrient intake is a major cause of insulin resistance, which leads to the onset and development of type 2 diabetes in cooperation with impaired pancreatic β cell function [1,2]. Therefore, insulin resistance has been paid a lot of attention as a therapeutic target for type 2 diabetes.

Skeletal muscle plays major roles in glucose uptake through glucose transporter in response to insulin action [2]. Accordingly, insulin resistance in skeletal muscle results in attenuated glucose tolerance. We showed in the previous report that the deficiency of AMPD1 which is highly selectively expressed in skeletal muscle attenuates insulin resistance induced by high fat diet feeding in C57BL6 mice that is an experimental model for insulin resistance [12]. Admyre et al. recently published that AMPD1 deficiency did not improve insulin resistance in C57BL6 mice [13]. They demonstrated that AMPD1 deficiency did not significantly augment, but at the very least did not attenuate glucose tolerance compared to control. The difference from our previous results may be due to the difference in experimental condition including the contents of feeding (5.08 kcal/g in our previous study vs. 5.24 kcal/g in Reference 13) and fasting time before glucose tolerance test (17 hours in our previous study vs. 4 hours in Reference 13).

To further clarify the underlying mechanism that contribute to the attenuated insulin resistance by AMPD1 deficiency in our experimental model, we studied insulin signaling in the present study and demonstrated that AMPD1 deficiency upregulates AMPK/Akt/mTOR/p70 S6 kinase pathway compared to control in skeletal muscle, but not in liver or white adipose tissue, among insulin-sensitive organs under high fat diet feeding. In accordance with this result, gene expression of PGC1 α and β that are downstream targets of p70 S6 kinase tended to increase in skeletal muscle [11]. Since these results suggest that AMPD1 deficiency inhibits the attenuation of insulin signaling in skeletal muscle under high fat diet feeding, they support the notion that AMPD1 deficiency attenuates insulin resistance induced by high fat diet feeding.

The protein complex mTORC1 and 2 that is composed of a kinase mTOR and associated proteins transduces signals of insulin and nutrients to a variety of downstream targets and serves as a central regulator of metabolism [14]. In this study, we showed that AMPD1 deficiency tended to increase the expression of mTOR and Raptor and promoted formation of mTORC1 in skeletal muscle after high fat diet challenge. In accordance with increased mTORC1 levels, a downstream target p70 S6 kinase showed enhanced activation by AMPD1 deficiency. Therefore, we suggest mTORC1-p70 S6 kinase pathway as a novel target of AMPD1 in skeletal muscle under high fat diet feeding. Further studies are needed to clarify the molecular mechanisms how AMPD1 levels and concomitantly modified cellular adenine nucleotide levels regulate the expression levels of mTOR and related molecules and the formation of mTORC1. Studies using in vitro culture system in combination with overexpression and knockdown of *Ampd1* gene and pharmacological intervention using AMP mimetics [15] could be useful for this purpose. We are currently studying in this context.

Although AMPD1 deficiency seems not to change gene expression of PGC1α and β, downstream targets of p70 S6 kinase that regulate mitochondria biogenesis for energy expenditure and glucose uptake in skeletal muscle [11], AMPD1 deficiency may modify the expression and function of other downstream targets for p70 S6 kinase, which include the factors that potentially contribute to insulin sensitivity regulation in skeletal muscle and the soluble factors including myokines that participate in inter-organ communication and regulate glucose metabolism in remote target organs [16]. Comprehensive analysis of transcriptome and proteome for skeletal muscle in combination with the comparison of the effects of diet type and AMPD1 deficiency are to be explored in the future research.

## Conclusion

We demonstrated that AMPD1 deficiency enhances activation of AMPK/Akt/mTORC1/p70 S6 kinase pathway in skeletal muscle under high fat diet feeding that could potentially contribute to attenuated insulin resistance, in accordance with our previous report using an *in vivo* experimental model [8]. Our current study provides novel evidence that mTORC1, a key regulator of insulin signal and cellular metabolism, and p70 S6 kinase are targets of AMPD1 in skeletal muscle under metabolic stress induced by high fat diet challenge.

## Abbreviations

AMP: Adenine monophosphate
AMPD: AMP Deaminase
AMPK: AMP-activated protein kinase
mTOR: Mammalian target of rapamycin
mTORC: Mammalian target of rapamycin complex
PGC1: Peroxisome proliferator-activated receptor-γ coactivator-1
WT: Wild type
