# Erratum to: AMPD1 regulates mTORC1-p70 S6 kinase axis in the control of insulin sensitivity in skeletal muscle

**DOI:** 10.1186/s12902-015-0078-2

**Published:** 2015-12-08

**Authors:** Andreas A. K. Tandelilin, Tetsuaki Hirase, Athanasius W. Hudoyo, Jidong Cheng, Keiko Toyama, Hiroko Morisaki, Takayuki Morisaki

**Affiliations:** Department of Bioscience and Genetics, National Cerebral and Cardiovascular Center Research Institute, 5-7-1 Fujishirodai, Suita, 565-8565 Osaka Japan; Department of Molecular Imaging in Cardiovascular Medicine, Osaka University Graduate School of Medicine, Suita, Osaka Japan; Department of Molecular Pathophysiology, Osaka University Graduate School of Pharmaceutical Sciences, Suita, Osaka Japan; Present address: Department of Internal Medicine, The First Affiliated Hospital of Shantou University Medical College, Shantou, 515031 Guangdong P R China

## Erratum

Unfortunately the original version of this article in *BMC Endocrine Disorders* [1] contained errors in the Figures. Several incorrect images were unintentionally taken as representative images in Fig. 1, 2, 3 and 4. Therefore the incorrect images have been replaced with the correct images below. The band intensity for these images has been re-measured and reanalysed, and the original findings have been reconfirmed. The conclusions of the article are unchanged.

**Fig. 1:**
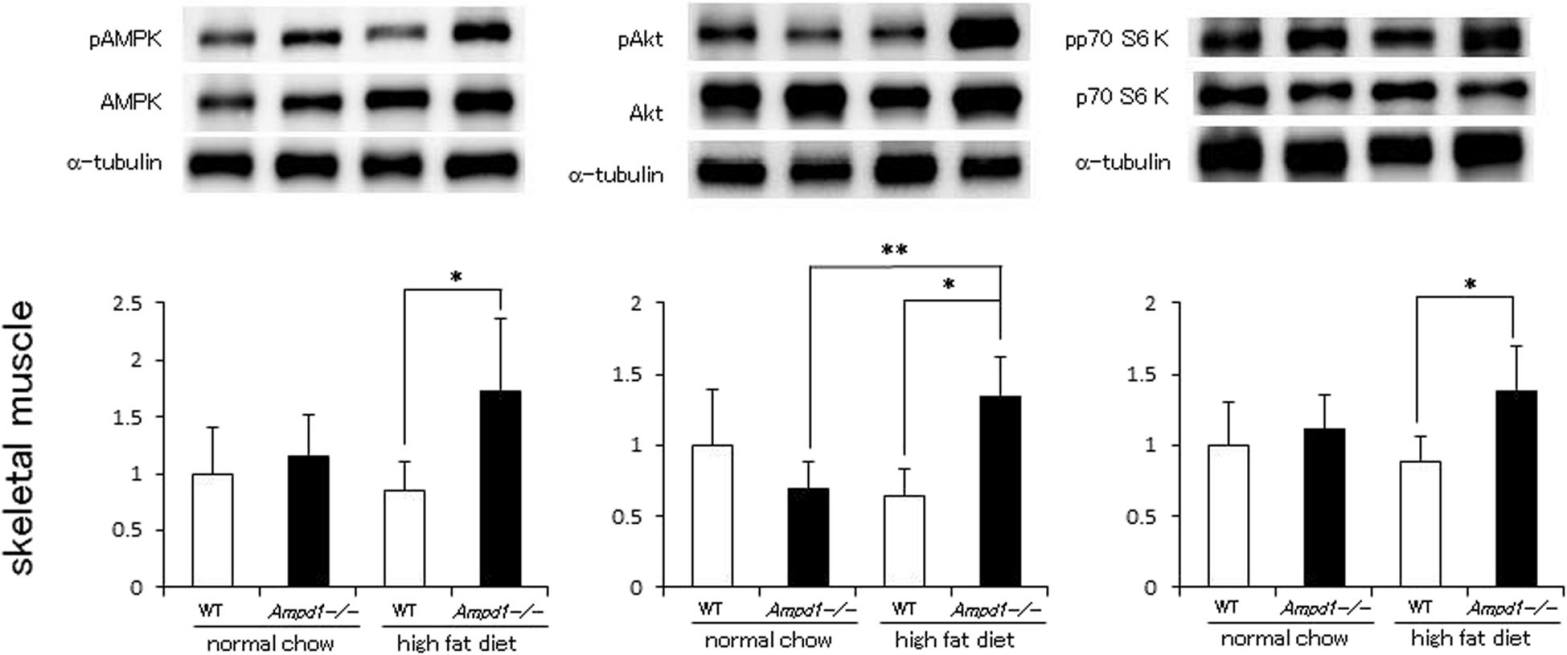
AMPD1 deficiency significantly augmented phosphorylation of AMPK, Akt and p70 S6 kinase after high fat diet challenge in skeletal muscles. Protein extracts from gastrocnemius muscle of wild type and AMPD1 deficient mice fed with normal chow and high fat diet were studied by immunoblotting for AMPK/phosphorylated AMPK (pAMPK), Akt/phosphorylated Akt (pAkt) and p70 S6 kinase/phosphorylated p70 S6 kinase (pp70 S6kinase) (*n* = 5 for each group). The ratio of the band intensity for phosphorylated form to that of total form measured as described in Methods was quantified and adjusted with that of α-tubulin. Representative immunoblot images are shown in upper panels. Data shown in lower panels are mean ± SD expressed relative to that of WT mice fed with normal chow. * and ** indicate *p <* 0.05 vs. WT mice fed with high fat diet and AMPD1-deficient mice fed with normal chow, respectively

**Fig. 2:**
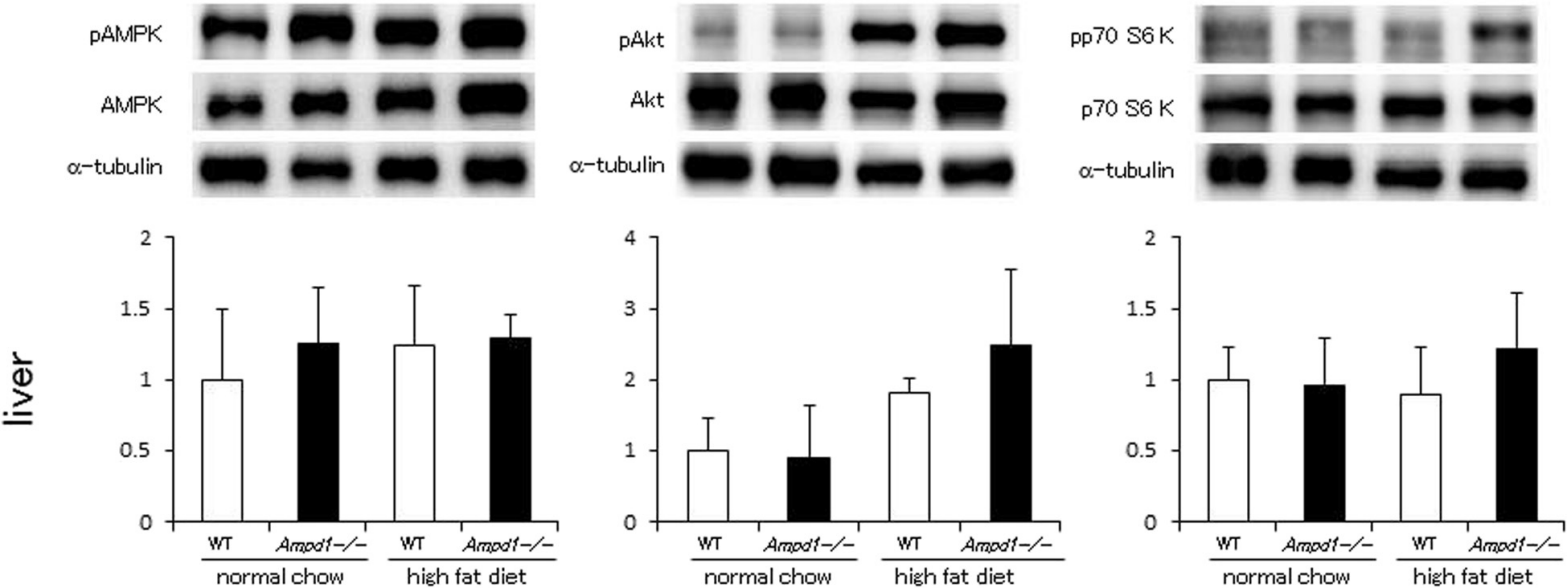
AMPD1 deficiency did not change phosphorylation levels of AMPK, Akt and p70 S6 kinase after high fat diet challenge in liver. Protein extracts from liver of wild type and AMPD1 deficient mice fed with normal chow and high fat diet were studied by immunoblotting for AMPK/phosphorylated AMPK (pAMPK), Akt/phosphorylated Akt (pAkt) and p70 S6 kinase/phosphorylated p70 S6 kinase (pp70 S6kinase) (*n* = 5 for each group). The ratio of the band intensity for phosphorylated form to that of total form was quantified and adjusted with that of α-tubulin. Representative immunoblot images are shown in upper panels. Data shown in lower panels are mean ± SD expressed relative to that of WT mice fed with normal chow

**Fig. 3:**
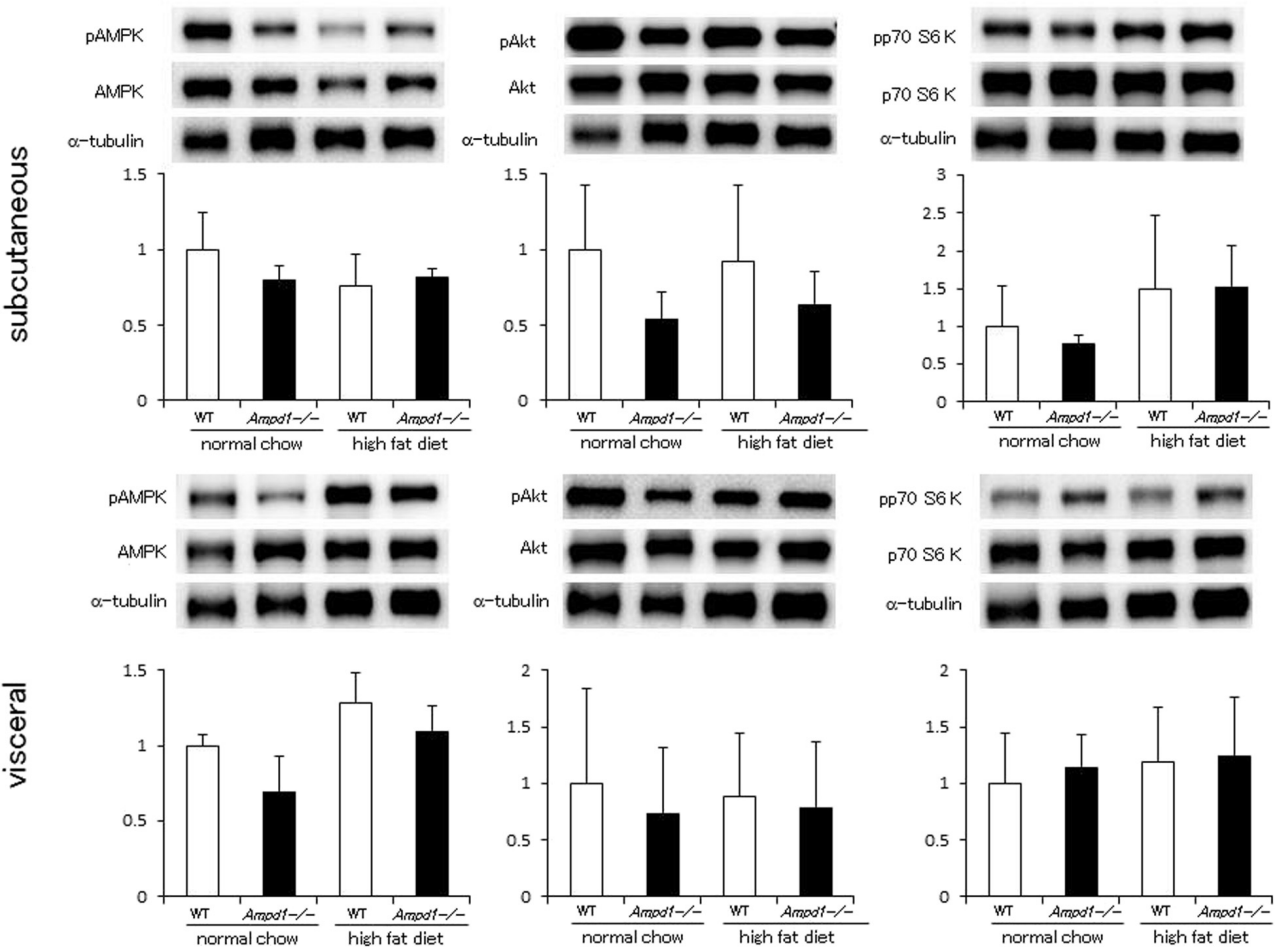
AMPD1 deficiency did not change phosphorylation levels of AMPK, Akt and p70 S6 kinase after high fat diet challenge in white adipose tissue. Protein extracts from subcutaneous and visceral adipose tissue of wild type and AMPD1 deficient mice fed with normal chow and high fat diet were studied by immunoblotting for AMPK/phosphorylated AMPK (pAMPK), Akt/phosphorylated Akt (pAkt) and p70 S6 kinase/phosphorylated p70 S6 kinase (pp70 S6kinase) (*n* = 5 for each group). The ratio of the band intensity for phosphorylated form to that of total form was quantified and adjusted with that of α-tubulin. Representative immunoblot images are shown in upper panels. Data shown in lower panels are mean ± SD expressed relative to that of WT mice fed with normal chow

**Fig. 4:**
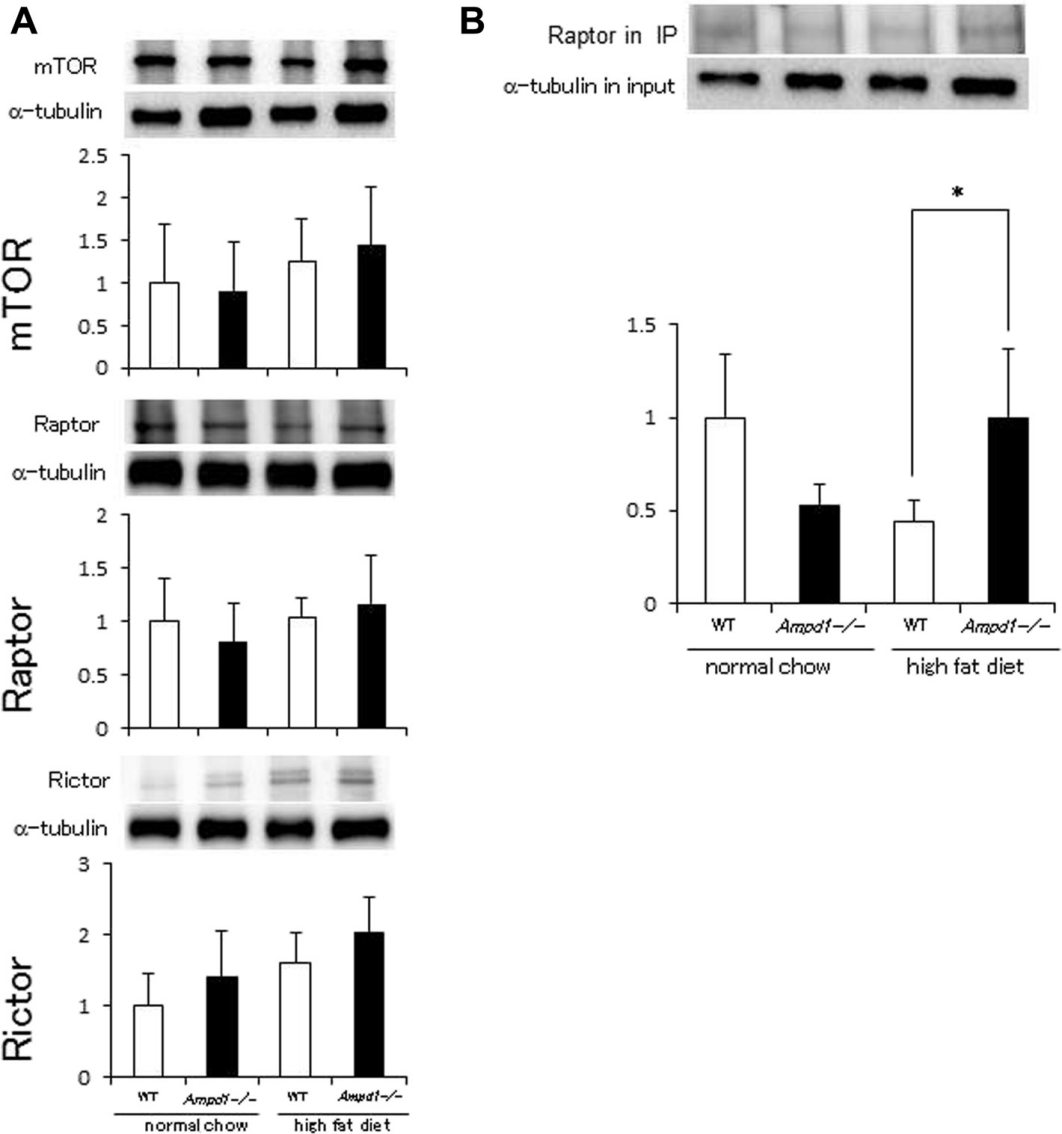
Promoted mTORC1 formation in skeletal muscle after high fat diet feeding by AMPD1 deficiency. **a** Protein extracts from gastrocnemius muscle of wild type and AMPD1 deficient mice fed with normal chow and high fat diet were subjected to immunoblotting for mTOR, Raptor and Rictor (*n* = 5 for each group). The band intensity was quantified and adjusted with that of α-tubulin. Representative immunoblot images are shown in upper panels. Data shown in lower panels are mean ± SD expressed relative to that of WT mice fed with normal chow. **b** mTOR immunoprecipitates from gastrocnemius muscle were analyzed by immunoblotting with anti-Raptor antibody. The band intensity of mTOR-bound Raptor in immunoprecipitates was adjusted with that of α-tubulin in the input lysates used for immunoprecipitation. Representative immunoblot images of Raptor in mTOR immunoporecipitates and α-tubulin in the input lysates are shown in upper panels. Data shown in lower panel are mean ± SD expressed relative to that of WT mice fed with normal chow (*n* = 5 for each group). * indicates *p* < 0.05 vs. WT mice fed with high fat diet

## Corrections

- Upper left panel of Fig. 1 (skeletal muscle)
  - The α-tubulin image in the representative image set for AMPK phosphorylation was replaced with the correct one.
- Upper right panel of Fig. 1 (skeletal muscle)
  - The α-tubulin image in the representative image set for p70 S6 kinase phosphorylation was replaced with the correct one.
- Middle graph of Fig. 1 (pAKT of skeletal muscle)
  - We obtained the significant difference between these conditions as shown in the previous version.
- Middle panel of Fig. 2 (liver)
  - The image set of pAkt, Akt and α-tubulin in Fig. 2, middle image panel was replaced with the correct one.
- Upper left panel of Fig. 3 (subcutaneous fat)
  - The α-tubulin image in the representative image set for AMPK phosphorylation was replaced with the correct one (Fig. 3), although the image of the latest version looks very similar to the previous version, but different from the previous version.
- Upper middle panel of Fig. 3 (subcutaneous fat)
  - The image set of pAkt, Akt and α-tubulin in Fig. 3 (upper middle panel) was replaced with the correct set.
- Upper right panel of Fig. 3 (subcutaneous fat)
  - The α-tubulin image in the representative image set for p70 S6 kinase phosphorylation was replaced with the correct one.
- Lower left panel of Fig. 3 (visceral fat)
  - The α-tubulin image in the representative image set for AMPK phosphorylation was replaced with the correct one.
- Lower right panel of Fig. 3 (visceral fat)
  - The image set of pp70 S6 K, p70 S6 K and α-tubulin in Fig. 3 (lower right panel) was replaced with the correct one.
- Figure 4a (Rictor)
  - The images of mTOR and α-tubulin corresponding to mTOR (upper), and α-tubulin corresponding to Raptor (middle) were replaced in Fig. 4a of the latest version. Also, the images of Rictor and the α-tubulin in the representative image set were replaced with the correct ones.
- Upper panel of Fig. 4b
  - The α-tubulin image in the representative image set for Raptor immunoprecipitation was replaced with the correct one, although the image of the latest version looks very similar to the previous version, but different from the previous version

## References

[1] Tandelilin AA, Hirase T, Hudoyo AW, Cheng J, Toyama K, Morisaki H, et al. AMPD1 regulates mTORC1-p70 S6 kinase axis in the control of insulin sensitivity in skeletal muscle. BMC Endocr Disord. 2015;15:11. 10.1186/s12902-015-0010-9 PMC4520000 25887856

